# Intersection of Sex and Frailty in Humoral Immune Responses to Influenza Vaccine in Community-Dwelling Older Adults

**DOI:** 10.1093/geroni/igaa057.869

**Published:** 2020-12-16

**Authors:** Janna Shapiro, Helen Kuo, Rosemary Morgan, Huifen Li, Sabra Klein, Sean Leng

**Affiliations:** 1 Johns Hopkins Bloomberg School of Public Health, Baltimore, Maryland, United States; 2 Johns Hopkins School of Medicine, Baltimore, Maryland, United States

## Abstract

Older adults bear the highest burden of severe disease and complications associated with seasonal influenza, with annual vaccination serving as the best option for protection. Variability in vaccine efficacy exists, yet the host factors that affect immune responses to inactivated influenza vaccines (IIV) are incompletely understood. We hypothesized that sex and frailty interact to affect vaccine-induced humoral responses among older adults. To test this hypothesis, community-dwelling adults above 75 years of age were recruited yearly, assessed for frailty (as defined by the Cardiovascular Health Study criteria), and vaccinated with the high-dose trivalent IIV. Humoral immune responses were evaluated via hemagglutination inhibition titers. The study began during the 2014-2015 influenza season, with yearly cohorts ranging from 76-163 individuals. A total of 617 vaccinations were delivered from 2014-2019. In preliminary analyses, the outcome of interest was seroconversion, defined as ≥ 4-fold rise in titers. Crude odds ratios suggest that females are more likely to seroconvert to influenza A strains (H1N1: OR = 1.39, (0.98-1.96) ; H3N2: 1.17 (0.85 – 1.62)), while males are more likely to seroconvert to the B strain (OR = 0.85 (0.60 – 1.22)). Furthermore, this sex difference was modified by frailty – for example, the odds of seroconversion to H1N1 were 65% higher for females than males among those who were nonfrail, and only 30% higher among females who were frail. Together, these results suggest that sex and frailty interact to impact immune responses to influenza vaccines. These findings may be leveraged to better protect vulnerable populations.

